# Predictors of early neurological deterioration in patients with acute ischemic stroke

**DOI:** 10.3389/fneur.2024.1433010

**Published:** 2024-08-21

**Authors:** Yang Zhou, Yufan Luo, Huazheng Liang, Zhenyu Wei, Xiaofei Ye, Ping Zhong, Danhong Wu

**Affiliations:** ^1^Emergency Department, Shaoxing People’s Hospital, Shaoxing, Zhejiang, China; ^2^Department of Neurology, Shanghai Fifth People’s Hospital, Fudan University, Shanghai, China; ^3^Suzhou Industrial Park Monash Research Institute of Science and Technology, Suzhou, Jiangsu Province, China; ^4^Southeast University-Monash University Joint Graduate School, Suzhou, Jiangsu Province, China; ^5^Monash University-Southeast University Joint Research Institute, Suzhou, Jiangsu Province, China; ^6^Department of Neurology, Shanghai Yangpu District Shidong Hospital, Shanghai, China; ^7^Department of Military Health Statistics, School of Health Service, People's Liberation Army, Naval Medical University, Shanghai, China

**Keywords:** outcome, early neurological deterioration, nomogram, ischemic stroke, predict

## Abstract

**Background:**

The present study aimed to develop a reliable and straightforward Nomogram by integrating various parameters to accurately predict the likelihood of early neurological deterioration (END) in patients with acute ischemic stroke (AIS).

**Methods:**

Acute ischemic stroke patients from Shaoxing People’s Hospital, Shanghai Yangpu District Shidong Hospital, and Shanghai Fifth People’s Hospital were recruited based on specific inclusion and exclusion criteria. The primary outcome was END. Using the LASSO logistic model, a predictive Nomogram was generated. The performance of the Nomogram was evaluated using the ROC curve, the Hosmer-Lemeshow test, and a calibration plot. Additionally, the decision curve analysis was conducted to assess the effectiveness of the Nomogram.

**Results:**

It was found that the Nomogram generated in the present study showed strong discriminatory performance in both the training and the internal validation cohorts when their ROC-AUC values were 0.715 (95% CI 0.648–0.782) and 0.725 (95% CI 0.631–0.820), respectively. Similar results were observed in two external validation cohorts when their ROC-AUC values were 0.685 (95% CI 0.541–0.829) and 0.673 (95% CI 0.545–0.800), respectively. In addition, CAD, SBP, neutrophils, TBil, and LDL were found to be positively correlated with the occurrence of END post-stroke, while lymphocytes and UA were negatively correlated.

**Conclusion:**

Our study developed a novel Nomogram that includes CAD, SBP, neutrophils, lymphocytes, TBil, UA, and LDL and it demonstrated strong discriminatory performance in identifying AIS patients who are likely to develop END.

## Introduction

Ischemic stroke (IS) represents the most common subtype of stroke and is a leading cause of mortality worldwide (1–3). Early neurological deterioration (END) refers to decline in neurological functions occurring within hours or days after acute ischemic stroke (AIS) onset. Previous studies have shown that the incidence of END varies from 5 to 40%, influenced by different inclusion criteria and assessment methods (4–6). Research has shown that END increases the risk of mortality and disability in AIS patients (7, 8). Due to the complex etiology and pathogenesis of END in AIS, there is still a lack of accurate and reliable early predictive markers, as well as effective prevention and treatment strategies. Consequently, it is imperative to investigate risk factors associated with END in AIS patients with an aim to minimize the occurrence of END.

Previous studies have tentatively used a number of predictors to identify END in AIS patients, such as hyperglycemia, baseline NIHSS score, and proximal artery occlusion (9, 10). Duan et al. found that elevated levels of HsCRP were independently correlated with END in AIS patients with atrial fibrillation (11). Furthermore, our previous study suggested that serum total bilirubin may be a potential biomarker for END in ischemic stroke patients (12). Despite these findings, an all-encompassing prognostic score that accurately predicts outcomes of AIS patient is still lacking.

Therefore, this study aimed to develop a Nomogram that integrated various parameters to accurately predict the likelihood of END in AIS patients.

## Materials and methods

### Subjects of the study

**Figure 1:**
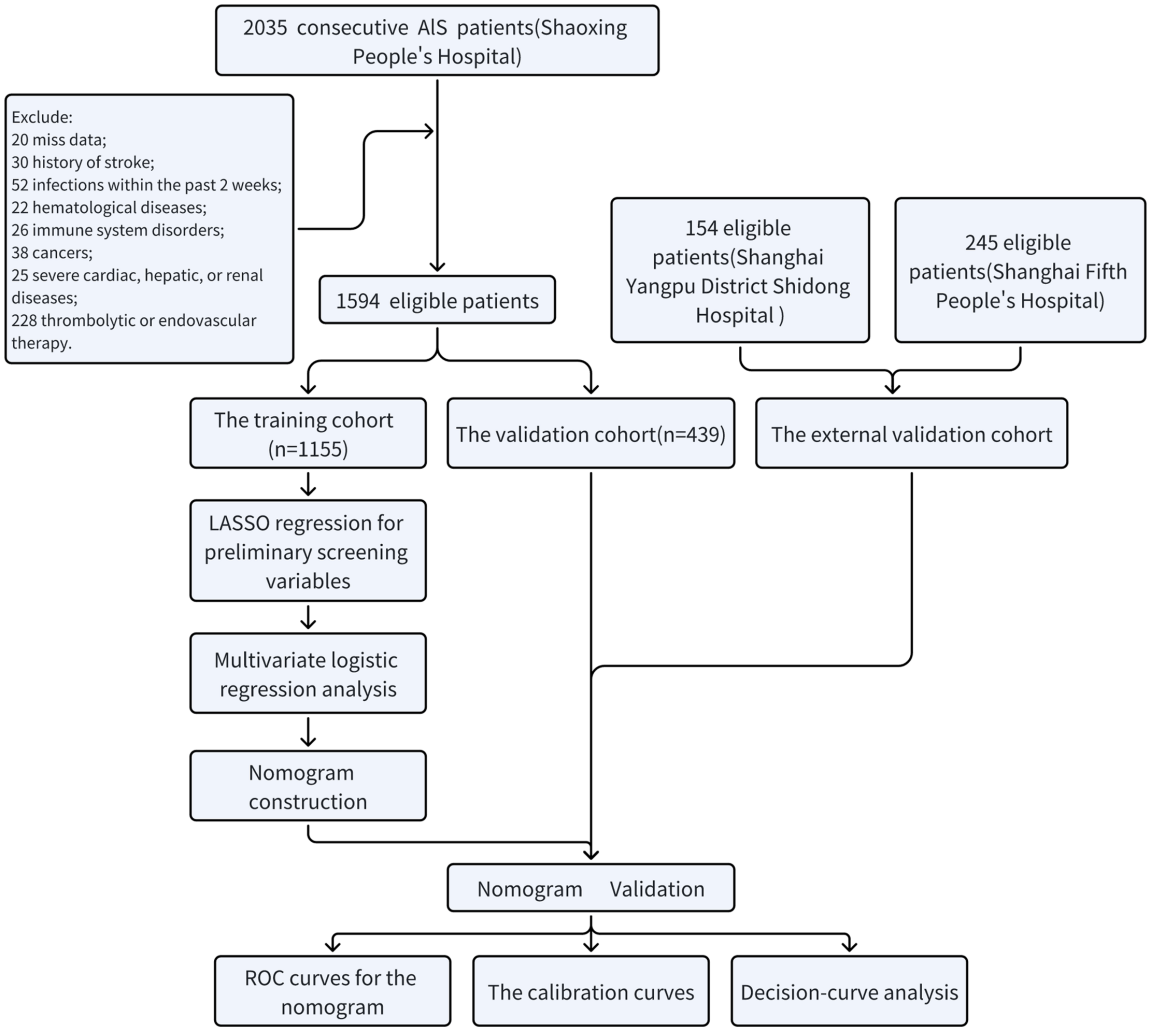
Flowchart for patient recruitment in the present study.

Acute ischemic stroke patients admitted to Shaoxing People’s Hospital from January 2018 to October 2023, Shanghai Yangpu District Shidong Hospital from January 2021 to October 2023, and Shanghai Fifth People’s Hospital from January 2018 to October 2020 were included in the present retrospective study. Inclusion criteria were: (1) aged ≥18 years; (2) completed brain computed tomography or magnetic resonance imaging scans during their hospitalization; (3) symptoms deteriorated within 48 h. Exclusion criteria were: (1) did not complete routine blood tests or were unable to obtain relevant parameters in the emergency department or on the day of admission; (2) history of stroke; (3) infections within the past 2 weeks; (4) patients with hematological diseases; (5) patients with immune system disorders; (6) patients with cancers; (7) patients with severe cardiac, hepatic, or renal diseases; (8) patients who received thrombolytic or endovascular therapy. Figure 1 showed the process of screening eligible participants. Participants in Shaoxing People’s Hospital were randomly allocated to the training and the internal validation cohorts in a 7:3 ratio (13, 14). Participants from Shanghai Yangpu District Shidong Hospital and Shanghai Fifth People’s Hospital were treated as external validation cohorts. They were referred to as the Shidong Hospital cohort and the Fifth Hospital cohort, respectively. This study was approved by the Ethics Committee of Shaoxing People’s Hospital (2021-KY-330-01), Shanghai Fifth People’s Hospital(2018 Ethics Approval NO.001), and Shanghai Yangpu District Shidong Hospital (2021-041-02).

### Data collection

Demographic information, physical examination, and laboratory findings were documented upon admission. Demographic information included age, gender, and smoking and drinking habits. Physical examination data included measurements of systolic blood pressure (SBP) and diastolic blood pressure (DBP). Laboratory findings included counts of white blood cells (WBC), neutrophils, lymphocytes, monocytes, platelets, levels of the C-reactive protein (CRP), total bilirubin (TBil), total cholesterol (TC), triglycerides (TG), low-density lipoprotein cholesterol (LDL), high-density lipoprotein cholesterol (HDL), apolipoprotein A (apoA), apolipoprotein B (apoB), uric acid (UA), creatinine, urea nitrogen (UN), fasting blood glucose (FBG), glycosylated hemoglobin (GHb), alanine aminotransferase (ALT), and the aspartate aminotransferase (AST). Additionally, information on the medication history (including anticoagulants, antihypertensives, antidiabetics, and statins) as well as comorbidities [such as history of coronary heart disease (CAD), hypertension, diabetes mellitus (DM), and atrial fibrillation (AF)] was collected. Neurological functions were assessed using the National Institutes of Health Stroke Scale (NIHSS) on the admission day and subsequently within the first 7 days by experienced clinicians. Ischemic stroke was classified into subtypes based on the criteria outlined in the Org 10,172 Acute Stroke Treatment Trial (15).

### Outcome assessment

The severity of stroke was assessed upon admission and subsequently 2–3 times daily for 7 days using the NIHSS score. All neurologists at each center received standardized training in NIHSS scoring and were blinded to the present study. Each participant underwent assessment by two certified neurologists from their assigned medical team. In cases of disagreement in NIHSS scoring, a third neurologist from each center was consulted to reach a final decision. Early neurological deterioration (END) was defined as an increase of ≥2 points in the NIHSS total score within 7 days of admission.

### Statistical analysis

The statistical analysis was performed using the R software version 3.6.2.^1^ Categorical variables were presented as counts and percentages [*n* (%)], whereas continuous variables were presented as medians with interquartile ranges (IQR). The independent sample *t*-test or Mann–Whitney U test was applied to compare continuous variables, and the Chi-square test or Fisher’s exact test was used to compare categorical variables. The LASSO regression analysis was conducted using the “Glmnet” software package to select the optimal subset of features. Based on the results of the LASSO regression analysis, a Nomogram was developed utilizing seven characteristic variables (CAD, SBP, neutrophils, lymphocytes, TBil, UA, and LDL). The performance of the Nomogram was then tested using data from the training cohort, internal validation cohort, and the external cohort. The discriminative performance of the Nomogram was evaluated by calculating the area under the receiver operating characteristic curve (AUC-ROC). Calibration was conducted by generating a calibration plot with 1,000 bootstrap resamples to assess the agreement between the actual and predicted outcomes. The Decision curve analysis was utilized to evaluate the clinical utility of the Nomogram. Statistically significant differences were indicated when *p* < 0.05.

## Results

### Baseline characteristics of subjects

Patients in Shaoxing People’s Hospital were categorized into END and non-END groups. The Univariate analysis revealed that age, baseline NIHSS score, CAD, AF, SBP, WBC, neutrophil, lymphocyte, CRP, TBil, TC, LDL, apoA, and UA were potentially associated with END (*p* < 0.05). Compared to patients in the non-END group, patients in the END group were older and had higher levels of the baseline NIHSS score, SBP, WBC, neutrophil, CRP, TBil, TC, LDL, apoA, CAD, and AF. In contrast, patients in the END group exhibited a lower count of lymphocytes and a lower level of UA (Table 1).

**Table 1:** Baseline characteristics of included patients.

Variable	END	Non-END	*p*
	(*n* = 106)	(*n* = 1,488)	
Age, years	73 [63,79]	70 [60,77]	0.027^*^
Male, *n* (%)	58 (54.7)	860 (57.8)	0.535
Drinking, *n* (%)	29 (27.4)	424 (28.5)	0.802
Smoking, *n* (%)	30 (28.3)	432 (29)	0.873
Baseline NIHSS score	3 [2,6]	2 [1,4]	<0.001^***^
TOAST			0.083
LAA, *n* (%)	47 (44.3)	513 (34.5)	
CE, *n* (%)	18 (17)	217 (14.6)	
SAO, *n* (%)	30 (28.3)	598 (40.2)	
Other, *n* (%)	11 (10.4)	160 (10.8)	
Hypertension, *n* (%)	76 (71.7)	1,008 (67.7)	0.399
DM, *n* (%)	27 (25.5)	377 (25.3)	0.975
CAD, *n* (%)	26 (24.5)	186 (12.5)	<0.001^***^
AF, *n* (%)	18 (17)	157 (10.6)	0.041^*^
Anticoagulant, *n* (%)	5 (4.7)	58 (3.9)	0.676
SBP, mmHg	159 [146,172]	148 [133,164]	<0.001^***^
DBP, mmHg	85.5 [78,97]	84 [76,93]	0.117
WBC, 10^9^	6.59 [5.59,8]	6.29 [5.14,7.53]	0.035^*^
Neutrophile, 10^9^	4.47 [3.43,6.2]	3.83 [2.99,5.01]	<0.001^***^
Lymphocyte, 10^9^	1.39 [1.03,1.7]	1.58 [1.24,2.01]	<0.001^***^
Monocyte, 10^9^	0.46 [0.37,0.58]	0.48 [0.38,0.59]	0.206
Platelet, 10^9^	199.5 [160,232]	203 [168,239.5]	0.433
CRP, mg/L	1.8 [0.74,6.79]	1.46 [0.68,4.28]	0.043^*^
TBil, μmol/L	15.1 [11.6,19.9]	12 [9.3,16.5]	<0.001^***^
TC, mmol/L	4.72 [3.96,5.15]	4.41 [3.76,5.08]	0.029^*^
TG, mmol/L	1.25 [0.91,2.01]	1.34 [0.99,1.84]	0.609
HDL, mmol/L	1.12 [0.93,1.37]	1.09 [0.93,1.29]	0.235
LDL, mmol/L	2.99 [2.36,3.36]	2.69 [2.17,3.26]	0.008^**^
apoA, g/L	1.18 [1.04,1.38]	1.17 [1.04,1.34]	0.282
apoB, g/L	0.95 [0.8,1.09]	0.89 [0.74,1.06]	0.047^*^
UN, μmol/L	4.72 [4.01,5.74]	4.89 [4.03,5.95]	0.199
UA, μmol/L	282.3 [231.4,347.7]	304.6 [251.8,370.4]	0.012^*^
Creatinine, μmol/L	67.9 [56.8,78.3]	68.7 [58.1,80.5]	0.491
FBG, mmol/L	5.52 [4.72,6.93]	5.26 [4.66,6.68]	0.171
GHb, %	6.15 [5.6,7]	6 [5.6,6.85]	0.501
ALT, U/L	16.1 [12.3,20.7]	17 [12.8,24.1]	0.123
AST, U/L	21 [17.9,26.4]	20.9 [17.4,25.7]	0.652

NIHSS, National Institute of Health Stroke Scale; TOAST, Trial of Org 10172 in acute stroke treatment; LAA, Large artery atherosclerosis; CE, Cardio-embolism; SAO, Small artery occlusion; DM, Diabetes mellitus; CAD, Coronary artery disease; AF, Atrial fibrillation; SBP, Systolic blood pressure; DBP, Diastolic blood pressure; WBC, White blood cell; CRP, C-Reactive protein; TBil, Total bilirubin; TC, Total cholesterol; TG, Total triglyceride; HDL, High density lipoprotein; LDL, Low density lipoprotein; apoA, Apolipoprotein A; apoB, Apolipoprotein B; UN, Urea nitrogen; UA, Uric acid; FBG, Fasting blood glucose; GHb, Glycosylated hemoglobin; ALT, Alanine aminotransferase; and AST, Aspartate aminotransferase.

^*^p < 0.05; ^**^p < 0.01; ^***^p < 0.001.

Patients in Shanghai Yangpu District Shidong Hospital were also divided into the END group and the non-END group. The Univariate analysis demonstrated that age, smoking, and hypertension were statistically significant factors associated with the occurrence of END (*p* < 0.05). Furthermore, compared to the non-END group, the END group exhibited an older age and a greater proportion of hypertension (Supplementary Table 1).

Patients in Shanghai Fifth People’s Hospital were divided into the END group and the non-END group as well. The Univariate analysis demonstrated that hypertension and the use of anticoagulant drugs were statistically significant factors associated with the occurrence of END (*p* < 0.05). The END group had a higher proportion of patients with hypertension and anticoagulant drug use compared to the non-END group (Supplementary Table 2).

### Baseline characteristics of the training and the validation cohorts

Patients in Shaoxing People’s Hospital were randomly assigned to the training and the internal validation cohorts in a ratio of 7:3. The training cohort consisted of 1,155 patients (median age: 70 years; 57.9% male), whereas the internal validation cohort consisted of 439 patients (median age: 69 years; 56.7% male). Table 2 displayed the demographic and clinical characteristics of the two cohorts. No significant difference in END was observed between the two cohorts (6.5 vs. 7.1%, *p* = 0.684). However, significant difference was found in the level of ApoB (*p* = 0.025) and the use of anticoagulant drugs (*p* = 0.028) between the cohorts, while no significant difference was observed in other variables.

**Table 2:** Baseline characteristics of the training and validation cohorts.

Variable	Training cohort	Validation cohort	*p*
	(*n* = 1,155)	(*n* = 439)	
Age, years	70 [60,77]	69 [59,77]	0.217
Male, *n* (%)	669 (57.9)	249 (56.7)	0.664
Drinking, *n* (%)	328 (28.4)	125 (28.5)	0.976
Smoking, *n* (%)	338 (29.3)	124 (28.2)	0.689
Baseline NIHSS score	2 [1,4]	2 [1,5]	0.783
TOAST			0.144
LAA, *n* (%)	404 (35)	156 (35.5)	
CE, *n* (%)	168 (14.5)	67 (15.3)	
SAO, *n* (%)	470 (40.7)	158 (36)	
Other, *n* (%)	113 (9.8)	58 (13.2)	
Hypertension, *n* (%)	787 (68.1)	297 (67.7)	0.853
DM, *n* (%)	288 (24.9)	116 (26.4)	0.542
CAD, *n* (%)	152 (13.2)	60 (13.7)	0.79
AF, *n* (%)	119 (10.3)	56 (12.8)	0.162
Anticoagulant, *n* (%)	38 (3.3)	25 (5.7)	0.028^*^
SBP, mmHg	149 [133,165]	149 [135,163]	0.867
DBP, mmHg	84 [76,94]	85 [76,93]	0.279
WBC, 10^9^	6.32 [5.14,7.51]	6.29 [5.22,7.69]	0.641
Neutrophile, 10^9^	3.85 [3,5.01]	3.9 [3.06,5.25]	0.364
Lymphocyte, 10^9^	1.56 [1.22,2]	1.57 [1.23,1.93]	0.904
Monocyte, 10^9^	0.48 [0.39,0.59]	0.47 [0.38,0.58]	0.325
Platelet, 10^9^	202 [167,237]	203 [172,246]	0.216
CRP, mg/L	1.46 [0.67,4.35]	1.53 [0.7,4.49]	0.508
TBil, μmol/L	12.2 [9.3,16.5]	12.1 [9.4,17]	0.856
TC, mmol/L	4.39 [3.73,5.08]	4.5 [3.83,5.15]	0.209
TG, mmol/L	1.33 [0.99,1.86]	1.34 [0.96,1.83]	0.936
HDL, mmol/L	1.09 [0.93,1.31]	1.09 [0.94,1.28]	0.889
LDL, mmol/L	2.68 [2.17,3.26]	2.77 [2.21,3.29]	0.175
apoA, g/L	1.17 [1.04,1.34]	1.17 [1.04,1.32]	0.649
apoB, g/L	0.89 [0.74,1.05]	0.92 [0.76,1.09]	0.025^*^
Urea, μmol/L	4.88 [4.01,5.9]	4.86 [4.05,6.04]	0.745
Uricacid, μmol/L	302.3 [248.9,369.5]	303.9 [256.9,368.8]	0.566
Creatinine, μmol/L	68.5 [58,79.9]	68.6 [57.7,81.4]	0.789
FBG, mmol/L	5.29 [4.66,6.67]	5.29 [4.71,6.93]	0.463
GHb, %	6 [5.6,6.8]	6 [5.6,7.1]	0.828
ALT, U/L	16.9 [12.8,23.6]	16.9 [12.3,24.1]	0.573
AST, U/L	21 [17.6,25.7]	20.6 [17.2,25.5]	0.182
END, *n* (%)	75 (6.5)	31 (7.1)	0.684

NIHSS, National Institute of Health Stroke Scale; TOAST, Trial of Org 10172 in acute stroke treatment; LAA, Large artery atherosclerosis; CE, Cardio-embolism; SAO, Small artery occlusion; DM, Diabetes mellitus; CAD, Coronary artery disease; AF, Atrial fibrillation; SBP, Systolic blood pressure; DBP, Diastolic blood pressure; WBC, White blood cell; CRP, C-Reactive protein; TBil, Total bilirubin; TC, Total cholesterol; TG, Total triglyceride; HDL, High density lipoprotein; LDL, Low density lipoprotein; apoA, Apolipoprotein A; apoB, Apolipoprotein B; UN, Urea nitrogen; UA, Uric acid; FBG, Fasting blood glucose; GHb, Glycosylated hemoglobin; ALT, Alanine aminotransferase; AST, Aspartate aminotransferase.

^*^p < 0.05.

Additionally, detailed baseline characteristics of patients from Shanghai Yangpu District Shidong Hospital and Shanghai Fifth People’s Hospital can be found in Supplementary Table 3. The median age of patients enrolled in the study at Shanghai Yangpu District Shidong Hospital was 62 years, and male patients accounted for 73.4%. The most common vascular risk factor observed was hypertension (42.9%), followed by DM (27.3%). A total of 16 patients with END were documented, representing a prevalence of 10.4%. In Shanghai Fifth People’s Hospital, the median age of enrolled patients was 68 years, and 68.2% were males. The predominant vascular risk factor observed was hypertension (65.7%), followed by DM (36.3%). A total of 18 patients of END were recorded, indicating a prevalence of 7.3% (Supplementary Table 3).

### The Univariate analysis of risk factors associated with END in the training cohort

The single-factor logistic regression analysis was conducted on risk factors associated with END within the training cohort. It was found that age (OR 1.02, 95% CI 1.002–1.042, *p* = 0.044), baseline NIHSS (OR 1.05, 95% CI 1.006–1.088, *p* = 0.027), CAD (OR 2.82, 95% CI 1.647–4.82, *p* < 0.001), AF (OR 1.93, 95% CI 1.025–3.622, *p* = 0.042), SBP (OR 1.02, 95% CI 1.006–1.026, *p* = 0.002), WBC (OR 1.11, 95% CI 1.012–1.212, *p* = 0.026), neutrophils (OR 1.17, 95% CI 1.069–1.275, *p* = 0.001), lymphocytes (OR 0.51, 95% CI 0.328–0.795, *p* = 0.003), TBil (OR 1.04, 95% CI 1.016–1.064, *p* = 0.001), LDL (OR 1.37, 95% CI 1.052–1.793, *p* = 0.02), and UA (OR 1, 95% CI 0.995–0.999, *p* = 0.038) were significantly associated with END (Table 3).

**Table 3:** Univariate analysis of risk factors associated with END in a training cohort.

Variable	B	SE	OR	CI	Z	*p*
Age	0.021	0.01	1.02	1.002–1.042	2.017	0.044^*^
Male	−0.084	0.241	0.92	0.573–1.475	−0.349	0.727
Drinking	−0.167	0.274	0.85	0.495–1.448	−0.608	0.543
Smoking	−0.138	0.27	0.87	0.513–1.479	−0.511	0.609
Baseline NIHSS	0.045	0.02	1.05	1.006–1.088	2.217	0.027^*^
TOAST	0.009	0.344	1.01	0.514–1.981	0.027	0.979
Hypertension	−0.007	0.256	0.99	0.601–1.64	−0.027	0.979
DM	−0.054	0.279	0.95	0.548–1.637	−0.194	0.847
CAD	1.036	0.274	2.82	1.647–4.82	3.786	<0.001^***^
AF	0.656	0.322	1.93	1.025–3.622	2.038	0.042^*^
Anticoagulant	0.55	0.543	1.73	0.598–5.024	1.014	0.311
SBP	0.016	0.005	1.02	1.006–1.026	3.089	0.002^*^
DBP	0.01	0.009	1.01	0.992–1.028	1.186	0.236
WBC	0.102	0.046	1.11	1.012–1.212	2.223	0.026^*^
Neutrophile	0.155	0.045	1.17	1.069–1.275	3.433	0.001^**^
Lymphocyte	−0.672	0.226	0.51	0.328–0.795	−2.973	0.003^**^
Monocyte	−0.503	0.698	0.6	0.154–2.374	−0.721	0.471
Platelet	−0.001	0.002	1	0.995–1.003	−0.37	0.711
CRP	0.007	0.007	1.01	0.993–1.021	1.015	0.31
TBil	0.039	0.012	1.04	1.016–1.064	3.181	0.001^**^
TC	0.178	0.103	1.19	0.976–1.462	1.719	0.086
TG	0.056	0.086	1.06	0.894–1.252	0.656	0.512
HDL	0.351	0.41	1.42	0.636–3.174	0.856	0.392
LDL	0.317	0.136	1.37	1.052–1.793	2.324	0.02^*^
apoA	0.438	0.506	1.55	0.575–4.176	0.865	0.387
apoB	−0.005	0.072	1	0.864–1.146	−0.065	0.948
UN	−0.051	0.07	0.95	0.829–1.09	−0.725	0.468
UA	−0.003	0.001	1	0.995–0.999	−2.071	0.038^*^
Creatinine	0	0.004	1	0.992–1.007	−0.121	0.904
FBG	0.053	0.044	1.05	0.967–1.149	1.203	0.229
GHb	0.052	0.078	1.05	0.904–1.228	0.668	0.504
ALT	0.002	0.005	1	0.992–1.011	0.282	0.778
AST	0.001	0.002	1	0.997–1.005	0.609	0.542

NIHSS, National Institute of Health Stroke Scale; TOAST, Trial of Org 10172 in acute stroke treatment; DM, Diabetes mellitus; CAD, Coronary artery disease; AF, Atrial fibrillation; SBP, Systolic blood pressure; DBP, Diastolic blood pressure; WBC, White blood cell; CRP, C-Reactive protein; TBil, Total bilirubin; TC, Total cholesterol; TG, Total triglyceride; HDL, High density lipoprotein; LDL, Low density lipoprotein; apoA, Apolipoprotein A; apoB, Apolipoprotein B; UN, Urea nitrogen; UA, Uric acid; FBG, Fasting blood glucose; GHb, Glycosylated hemoglobin; ALT, Alanine aminotransferase; AST, Aspartate aminotransferase.

^*^p < 0.05; ^**^p < 0.01; ^***^p < 0.001.

### The LASSO regression analysis

**Figure 2:**
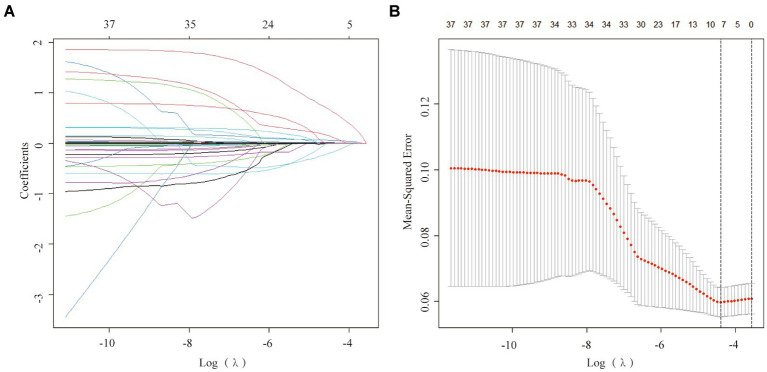
Factor selection using the least absolute shrinkage and selection operator (LASSO) logistic regression. **(A)** The LASSO coefficient profiles of the candidate variables. The binomial deviance is plotted versus log (λ). **(B)** Tuning parameter (λ) selection in the LASSO logistic regression performed using 10-fold cross-validation via the minimum criteria.

To mitigate potential multicollinearity among variables, the LASSO regression analysis was utilized to identify key variables (Figure 2A). This employed 10-fold cross-validation to select the Lambda parameter that minimized the mean square error, which yielded the optimal model (Figure 2B). Seven significant variables were identified: CAD, SBP, neutrophils, lymphocytes, TBil, UA, and LDL, with a Lambda value of 0.01233904.

The Multivariable logistic regression analysis was performed on these seven characteristic variables obtained from the LASSO regression analysis (Table 4). After adjusting all potential confounders, CAD (OR 3.09, 95% CI 1.732–5.505, *p* < 0.001), SBP (OR 1.01, 95% CI 1–1.023, *p* = 0.038), lymphocytes (OR 0.63, 95% CI 0.402–0.976, *p* = 0.039), UA (OR 1, 95% CI 0.995–0.999, *p* = 0.034), and LDL (OR 1.51, 95% CI 1.132–2.022, *p* = 0.005) were identified as independent predictors of END in this model (Table 4).

**Table 4:** Multivariate logistic regression analysis.

Characteristics	B	SE	OR	CI	*Z*	*p*
CAD	1.127	0.295	3.09	1.732–5.505	3.821	<0.001^***^
SBP	0.011	0.006	1.01	1–1.023	2.076	0.038^*^
Neutrophil	0.087	0.049	1.09	0.991–1.2	1.752	0.08
Lymphocyte	−0.467	0.226	0.63	0.402–0.976	−2.064	0.039^*^
TBil	0.025	0.013	1.03	1–1.052	1.872	0.061
UA	−0.003	0.001	1	0.995–0.999	−2.117	0.034^*^
LDL	0.414	0.148	1.51	1.132–2.022	2.788	0.005^**^

CAD, Coronary artery disease; SBP, Systolic blood pressure; TBil, Total bilirubin; UA, Uric acid; LDL, Low density lipoprotein.

^*^p < 0.05; ^**^p < 0.01; ^***^p < 0.001.

### Construction of the nomogram

**Figure 3:**
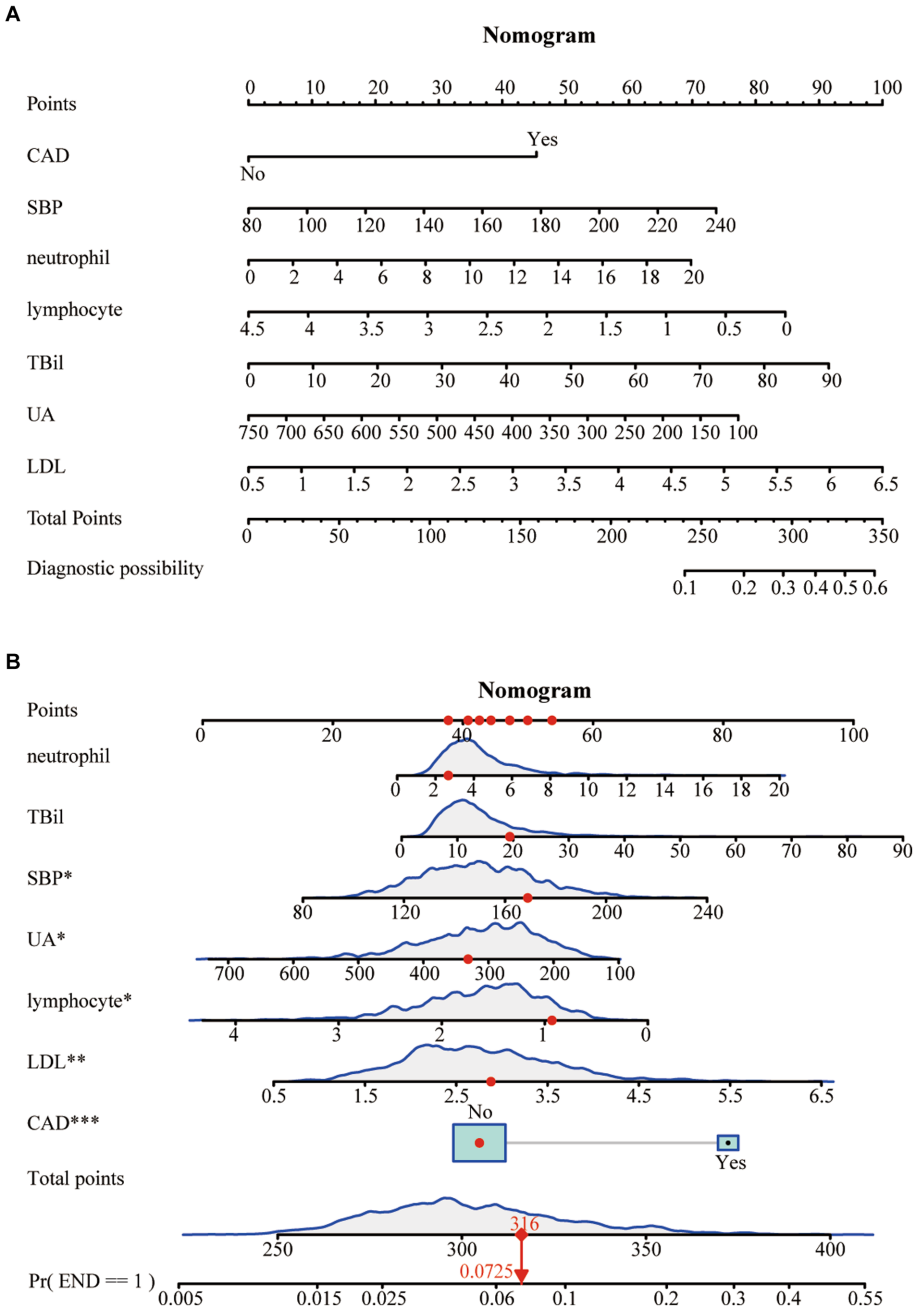
**(A)** A risk prediction model for poor outcomes. **(B)** An example of using the Nomogram.

A Nomogram was constructed based on the seven characteristic variables (CAD, SBP, neutrophils, lymphocytes, TBil, UA, and LDL) identified by the LASSO regression analysis. It predicted the probability of END by assigning scores to each independent predictor on a scale from 0 to 100. Higher cumulative scores on the Nomogram indicated an elevated risk of END, whereas lower scores indicated a reduced probability (Figure 3A). For example, when the values for neutrophils = 2.7*10^9^, total bilirubin = 20 μmoL/L, SBP = 170 mmHg, uric acid = 320 μmoL/L, lymphocytes = 0.9*10^9^, LDL = 2.9 mmoL/L, and no history of CAD were entered, the estimated risk of END was 7.25% (Figure 3B).

### Validation of the nomogram in the training and the validation cohorts

**Figure 4:**
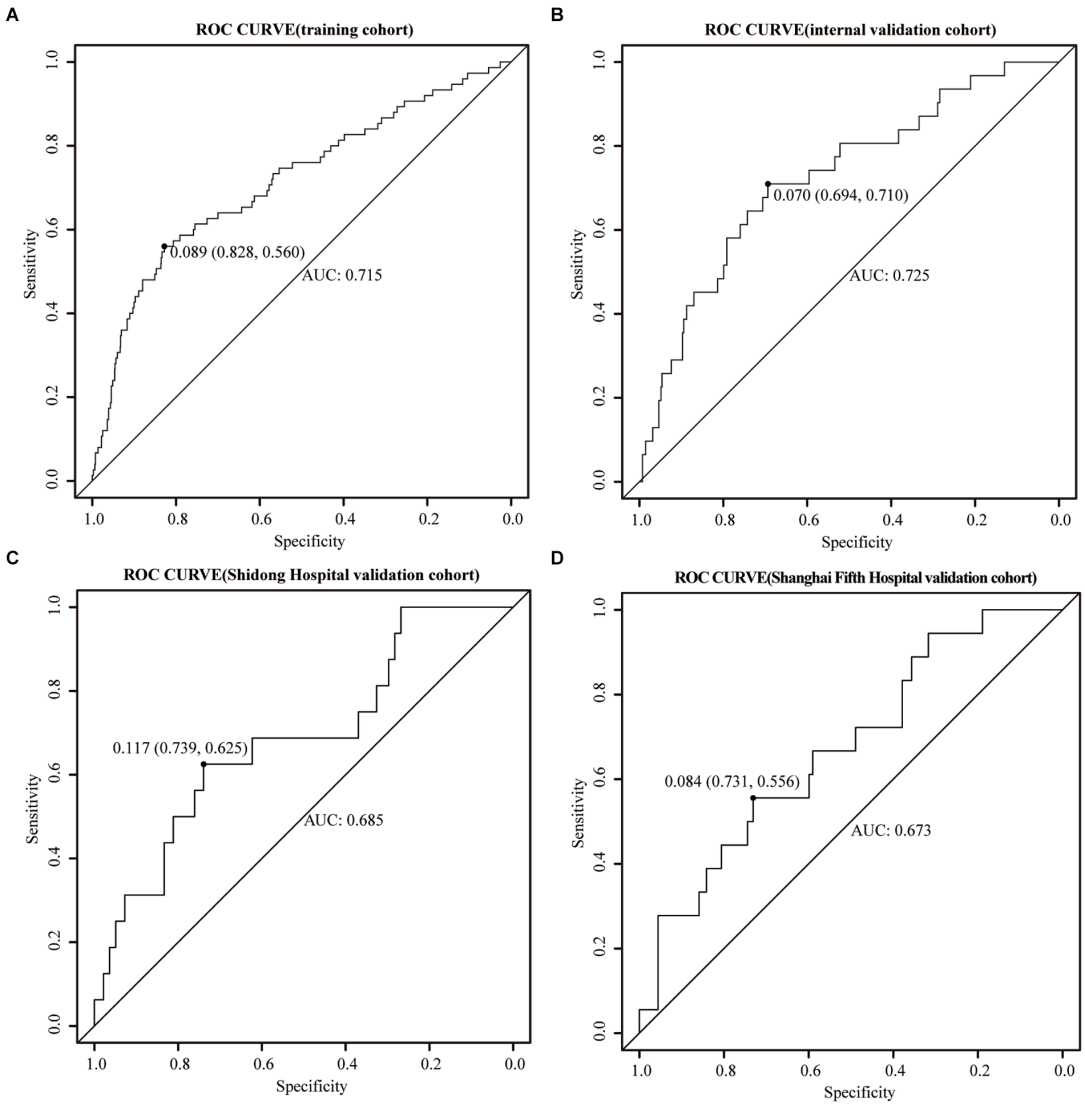
ROC curves were generated for the Nomogram in the training cohort **(A)**, the internal validation cohort **(B)**, the Shidong Hospital validation cohort **(C)**, and the Shanghai Fifth Hospital validation cohort **(D)**.

**Figure 5:**
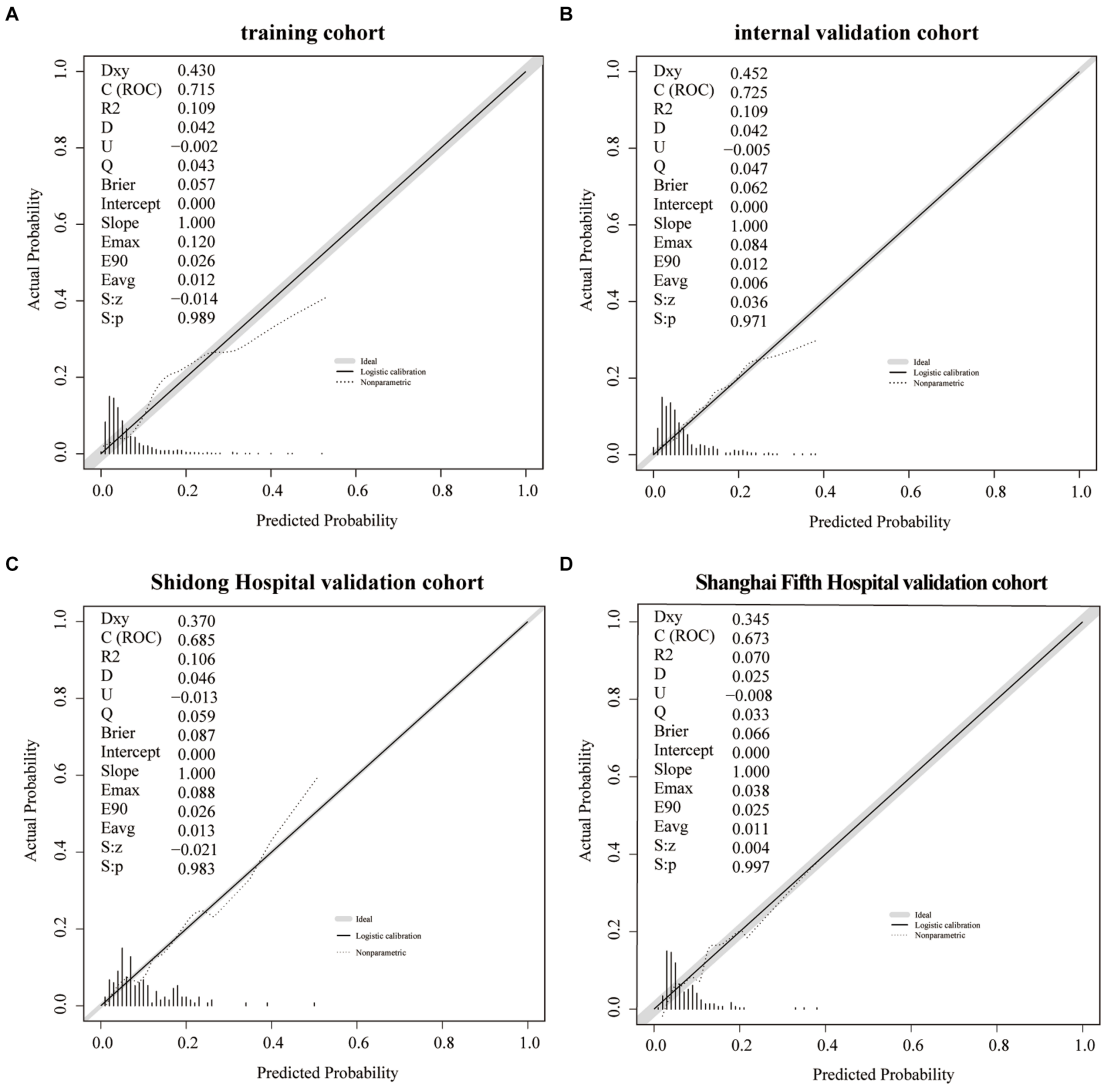
A calibration plot of the Nomogram in the training cohort **(A)**, the internal validation cohort **(B)**, the Shidong Hospital validation cohort **(C)**, and the Shanghai Fifth Hospital validation cohort **(D)**.

The training cohort demonstrated a Hosmer-Lemeshow χ^2^ of 8.14 (*p* = 0.519), with an area under the ROC curve of 0.715 (95% CI 0.648–0.782) (Figure 4A). In the internal validation cohort, the Hosmer-Lemeshow χ^2^ was 4.457 (*p* = 0.879), and the area under the ROC curve was 0.725 (95% CI 0.631–0.820) (Figure 4B). Furthermore, in the patient cohorts enrolled at Shanghai Yangpu District Shidong Hospital, the Hosmer-Lemeshow χ^2^ was 12.02 (*p* = 0.212), with an area under the ROC curve of 0.685 (95% CI 0.541–0.829) (Figure 4C). In the patient cohort enrolled in Shanghai Fifth People’s Hospital, the Hosmer-Lemeshow χ^2^ was 6.86 (*p* = 0.652), with an area under the ROC curve of 0.673 (95% CI 0.545–0.800) (Figure 4D). These findings further demonstrated strong calibration and discrimination. Calibration plots, comparing the predicted probability of END using the Nomogram to the observed probability of END, revealed a significant prediction accuracy in all the cohorts (Figure 5).

**Figure 6:**
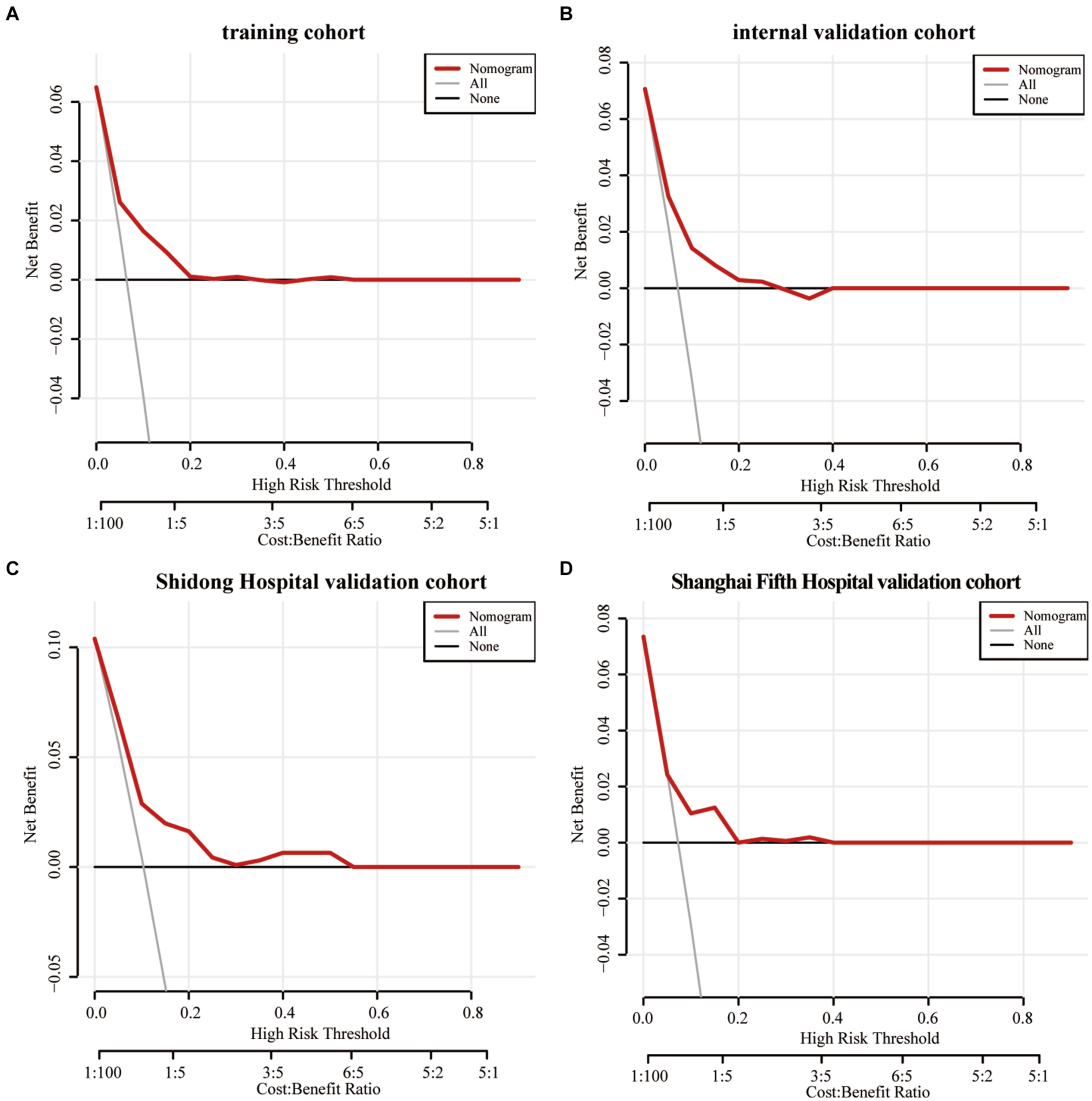
The decision curve analysis of the Nomogram of the training cohort **(A)**, the internal validation cohort **(B)**, the Shidong Hospital validation cohort **(C)**, and the Shanghai Fifth Hospital validation cohort **(D)**.

The Decision curve analysis was performed on the Nomogram to evaluate its predicability for END and to determine whether the model provides a greater net benefit. The Nomogram showed that when the risk thresholds ranged from 0 to 0.2 in the training cohort (Figure 6A), from 0 to 0.25 in the internal validation cohort (Figure 6B), from 0 to 0.55 in the Shanghai Yangpu District Shidong Hospital validation cohort (Figure 6C) and from 0 to 0.4 in the Shanghai Fifth People’s Hospital validation cohort (Figure 6D), adopting the strategy with the greatest net benefit ensured that all AIS patients would choose this strategy, thus ensuring that no AIS patient would miss out on it. At a risk threshold of 0.1 for AIS patients, net benefits of 2, 1.6, 2.5, and 1% in the training and validation cohorts, respectively, were yielded (Figure 6).

### Comparison between different indicators in the nomogram

The ROC analysis was performed for each indicator in the Nomogram. The results demonstrated that the AUC of neutrophils was larger than that of other indicators in the internal validation cohort (AUC = 0.653, 95%CI 0.556–0.750) and Shanghai Yangpu District Shidong Hospital validation cohort (AUC = 0.658, 95%CI 0.561–0.754). Conversely, in the Shanghai Fifth People’s Hospital validation cohort, the total bilirubin AUC surpassed other indicators (AUC = 0.628, 95%CI 0.500–0.756) (Supplementary Figure 1).

## Discussion

The present study introduced a novel Nomogram that included CAD, SBP, neutrophils, lymphocytes, TBil, UA, and LDL as predictors for END in AIS patients. These variables are routinely collected in the clinical setting; therefore, the Nomogram is practical for risk stratification. ROC curves and calibration plots were used in both the training and validation cohorts to evaluate the discrimination and calibration performance of the Nomogram. The results demonstrated ROC-AUC values of 0.715 (95% CI 0.648–0.782) and 0.725 (95% CI 0.631–0.820) in the training and the internal validation cohorts, respectively, signifying strong discriminatory performance. The external validation cohorts also exhibited ROC-AUC values of 0.685 (95% CI 0.541–0.829) and 0.673 (95% CI 0.545–0.800), indicating good discriminatory performance as well. Additionally, calibration curves provided further evidence on the model’s reliable calibration capacity. Importantly, CAD, SBP, neutrophil, TBil, and LDL were positively correlated with END occurrence post-stroke, while lymphocytes and UA were negatively correlated.

Previous studies have constructed other models to predict the risk of developing END in AIS patients. However, their scoring systems were limited in the ability to predict END using a small number of parameters. Wang et al. (16) developed a scoring system to predict END based on MRI-derived radiomics and clinical metrics. However, MRI examination is expensive and time-consuming, which may negatively impact the timing of diagnosis and therapy and increase the probability of END. Xie et al. (17) developed a prediction model consisting of the initial NIHSS score, middle cerebral artery stenosis, and carotid stenosis ≥50% to predict END in AIS patients. However, this study was conducted in a single center and included a relatively small number of patients. In contrast, our multi-center study effectively addressed these concerns by analyzing a larger patient cohort, which enhanced the reliability of the findings.

Coronary heart diseases, hypertension, and ischemic stroke often simultaneously occur and share common risk factors. Previous studies have shown that patients with ischemic stroke may experience clinical symptoms of coronary artery diseases, and effective management of coronary heart diseases might potentially reduce the risk of ischemic stroke (18). A substantial 10-year study conducted in China further substantiated the notion that coronary heart diseases may independently predict the recurrence of stroke (19). Our study further reinforces this concept by highlighting that coronary heart diseases independently predict END in AIS patients. Hypertension is a significant risk factor of AIS and can lead to more severe neurological deficits, worsening functional outcomes of AIS patients. This is likely attributed to the complex interplay between cerebrovascular autoregulation, neuronal activity, and brain bioenergetics (20). Turana et al. (21) reported a positive correlation between systolic blood pressure (SBP) and the incidence of stroke. In many Asian countries, adherence to hypertension treatment is crucial for stroke prevention. A cohort study by Zhou et al. (22) identified SBP as a risk factor for predicting the recurrence of ischemic stroke, which is consistent with our data demonstrating a strong correlation between SBP and END in patients with AIS. Therefore, effective management of coronary heart diseases and blood pressure has the potential to mitigate adverse outcomes in patients with AIS.

Oxidative stress, inflammatory response, and cholesterol accumulation collectively contribute to the progression of stroke (23). Studies have shown that ischemia–reperfusion injury generates a significant amount of oxygen free radicals, which result in neuronal oxidative stress and lead to compromised functions of cellular components including proteins, phospholipids, and nucleic acids. Such damage also impacts the structure of mitochondria and triggers the release of excitatory amino acids, ultimately accelerating neuronal necrosis (24). Post-ischemic brain injury activates the nuclear factor κB, which upregulates the expression of inflammatory factors, chemokines, and cell adhesion molecules. This cascade of events recruits inflammatory cells to the site of injury, resulting in microvascular occlusion, generation of oxygen free radicals, release of cytotoxic enzymes, and vasomotor changes that exacerbate post-ischemic brain injury (25, 26).

Uric acid, an essential endogenous antioxidant, plays a crucial role in protecting the brain from oxidative injury by inhibiting the accumulation of reactive oxygen species and the formation of nitrotyrosine (27). Animal studies have shown that high concentrations of uric acid can ameliorate brain tissue injury and the production of reactive oxygen species in a rat model of middle cerebral artery occlusion (28). Furthermore, a prospective study involving 881 AIS patients revealed an inverse relationship between neurological impairment, lesion volume on admission, and the level of uric acid (29). Our study supports these findings by demonstrating a negative association between the concentration of uric acid and the occurrence of END in AIS patients. Some researchers proposed that neutrophils may enhance the immune response in patients with ischemic stroke by producing pro-inflammatory cytokines (30) and triggering the expression of matrix metalloproteinase-9 (MMP-9), thereby contributing to the disruption of the blood–brain barrier and exacerbating brain damage (31). Conversely, lymphocytes are thought to have a protective role in inflammatory response to ischemic injury (32). A decrease in lymphocytes may indicate a stress response associated with cortisol, potentially increasing the production of pro-inflammatory cytokines (33). Our study demonstrated that neutrophils and lymphocytes were independent risk factors for END after AIS, with neutrophils positively correlated and lymphocytes negatively correlated with END occurrence. These findings are consistent with those of a previous study (34). Additionally, bilirubin can activate microglia and induce the release of inflammatory factors, such as tumor necrosis factor alpha (TNF-α), interleukin 1β, and interleukin 6, thus promoting neurotoxicity (35, 36). A study showed a positive correlation between the level of the serum bilirubin and the NIHSS, suggesting that higher concentrations of the serum bilirubin were associated with more severe stroke, which may be served as useful markers to reflect the severity of illness (37). Our findings also support this, showing a positive correlation between total bilirubin and the occurrence of END in AIS patients. Higher plasma levels of LDL result in increased adhesion of circulating monocytes to arterial endothelial cells, promoting LDL entry into the vascular intima (38). This process can cause damage to endothelial cells, formation of foam cells, activation of cell surface receptors on monocytes and vascular smooth muscle cells, initiation of inflammation, and promotion of AIS development (38, 39). A meta-analysis showed that for each 1 mmol/L decrease in LDL-C, there was a 17% decrease in the incidence of stroke (40, 41). A study by Amarenco et al. investigated the target level of LDL cholesterol for secondary prevention of cerebrovascular events. They found that maintaining the level of LDL below 1.8 mmol/L in patients with ischemic stroke or transient ischemic attack and evidence of atherosclerosis was associated with a lower risk of vascular events compared to those with their LDL between 2.3 and 2.8 mmol/L (42). Our study further confirmed that LDL is an independent predictor of END in AIS patients.

The Decision curve analysis (DCA) is a novel approach for evaluating the performance of predictive models, such as nomograms (43, 44). The results of the DCA in this study demonstrated the efficacy of nomograms in predicting END and illustrating the overall benefit of clinical outcomes at various threshold probabilities. A net benefit of zero in DCA indicates that no treatment would be required for AIS patients. AIS patients at a higher risk of END may benefit from additional interventions, including endovascular therapy, hematoma resection, cranial decompression, or specific medications. Results of the DCA supported the idea that the Nomogram can effectively identify high-risk patients who may develop END.

To the best of our knowledge, there is limited research conducted in a multicenter format exploring the predictive role of the Nomogram in assessing END in AIS patients. In this study, we developed a new Nomogram model specifically designed to predict END in AIS patients. The Nomogram model exhibited excellent predictive performance and accuracy. Furthermore, the Nomogram prediction model identified key clinical factors that can be utilized for early prediction of END in AIS patients, thereby maximizing clinical benefits.

However, this study has a number of limitations that should be acknowledged. Firstly, being a retrospective study, it is susceptible to selection and recall bias. Secondly, certain inflammatory biomarkers, such as interleukin 6 and tumor necrosis factor α, were not measured in this study and will be evaluated in future research. Furthermore, the participants were exclusively from East China, it is challenging to extrapolate our findings to other populations. Therefore, it is essential to test the Nomogram in other populations to ensure its generalizability beyond China.

## Conclusion

Our study developed a novel Nomogram that included CAD, SBP, neutrophils, lymphocytes, TBil, UA, and LDL to predict the risk of developing END in AIS patients. The number of neutrophils and the level of total bilirubin were strong predictors of END. Future large-scale studies are needed to improve the accuracy of the Nomogram model in predicting END in AIS patients.

## Data Availability

The raw data supporting the conclusions of this article will be made available by the authors without undue reservation.

## Footnotes

^1^https://www.r-project.org/

## Supplementary material

SUPPLEMENTARY FIGURE 1 ROC curves were generated to evaluate the performance of different indicators within the Nomogram using the internal validation cohort **(A)**, the Shidong Hospital validation cohort **(B)**, and the Shanghai Fifth Hospital validation cohort **(C)**.
