# Supplementary figures and images for: Predictors of early neurological deterioration in patients with acute ischemic stroke
Source: Front Neurol. 2024 Aug 21;15:1433010. doi: 10.3389/fneur.2024.1433010 (PMC11371773; doi:10.3389/fneur.2024.1433010)

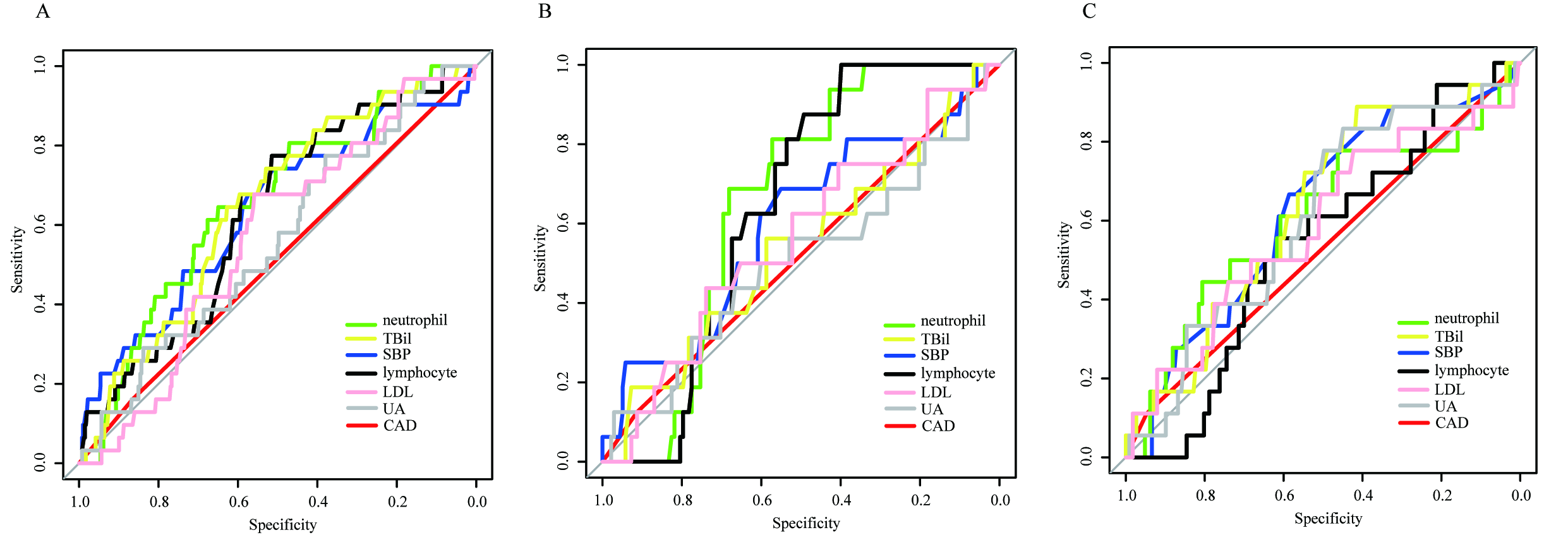

Supplement: SUPPLEMENTARY FIGURE 1 — ROC curves were generated to evaluate the performance of different indicators within the Nomogram using the internal validation cohort (A), the Shidong Hospital validation cohort (B), and the Shanghai Fifth Hospital validation cohort (C). [file Image_1.TIF]
