# Supplementary material for: Predictors of early neurological deterioration in patients with acute ischemic stroke
Source: Front Neurol. 2024 Aug 21;15:1433010. doi: 10.3389/fneur.2024.1433010 (PMC11371773; doi:10.3389/fneur.2024.1433010)
Supplement: Supplementary file 2 [file Table_1.DOCX]

Supplementary Table 1 Baseline characteristics of included patients at Shidong Hospital

| Variable | END | Non-END | P |
| --- | --- | --- | --- |
|  | (n=16) | (n=138) |  |
| Age, years | 73.5[61.5,77] | 61[53,68] | 0.014^*^ |
| Male, n(%) | 10(62.5) | 103(74.6) | 0.298 |
| drinking, n(%) | 5(33.3) | 60(43.5) | 0.348 |
| smoking, n(%) | 11(68.8) | 58(42) | 0.042^*^ |
| Baseline NIHSS score | 6.5[2.5,11] | 5[3,10] | 0.778 |
| TOAST |  |  | 0.901 |
| LAA, n(%) | 6(40) | 56(40.6) |  |
| CE, n(%) | 3(20) | 29(21) |  |
| SAO, n(%) | 6(40) | 49(35.5) |  |
| other, n(%) | 1(6.7) | 4(2.9) |  |
| hypertension, n(%) | 11(68.8) | 55(39.9) | 0.027^*^ |
| DM, n(%) | 5(33.3) | 37(26.8) | 0.706 |
| CAD, n(%) | 2(13.3) | 12(8.7) | 0.616 |
| AF, n(%) | 3(20) | 29(21) | 0.833 |
| anticoagulant, n(%) | 2(13.3) | 8(5.8) | 0.262 |
| SBP, mmHg | 156[142.5,172.5] | 148[134,164] | 0.203 |
| DBP, mmHg | 84.5[77.5,96] | 83[76,95] | 0.655 |
| WBC, 109 | 8.48[6.82,9.93] | 7.77[6.16,9.3] | 0.414 |
| neutrophile, 109 | 6.24[4.83,7.90] | 5.66[3.98,7.35] | 0.205 |
| lymphocyte, 109 | 1.38[1.20,1.57] | 1.55[1.09,2.11] | 0.203 |
| monocyte, 109 | 0.42[0.36,0.49] | 0.5[0.41,0.61] | 0.107 |
| platelet, 109 | 191[163,220] | 189[160,214] | 0.642 |
| CRP, mg/L | 1.83[0.56,4.11] | 1.42[0.72,3.76] | 0.84 |
| TBil, μmol/L | 13.8[11.3,19.2] | 15.2[11.7,19] | 0.651 |
| TC, mmol/L | 4.98[3.79,5.49] | 4.92[4.14,5.63] | 0.795 |
| TG, mmol/L | 1.01[0.74,1.8] | 1.26[0.82,1.91] | 0.554 |
| HDL, mmol/L | 1.24[1.03,1.44] | 1.15[0.99,1.32] | 0.386 |
| LDL, mmol/L | 2.59[2.15,3.29] | 2.84[2.29,3.41] | 0.417 |
| apoA, g/L | 1.39[1.27,1.6] | 1.26[1.12,1.49] | 0.13 |
| apoB, g/L | 0.99[0.75,1.07] | 0.97[0.79,1.16] | 0.58 |
| UN, μmol/L | 4.51[3.81,5.57] | 5.05[4.19,5.99] | 0.217 |
| UA, μmol/L | 331[278.5,418] | 342[298,401] | 0.948 |
| creatinine, μmol/L | 72.5[57.5,91] | 69[61,79] | 0.655 |
| FBG, mmol/L | 6.35[5.66,7.25] | 5.75[5,7.1] | 0.169 |
| GHb, % | 5.95[5.35,6.45] | 5.7[5.3,6.4] | 0.747 |
| ALT, U/L | 17.5[14,24.5] | 21[16,30] | 0.132 |
| AST, U/L | 18.5[15,34] | 20[16,25] | 0.953 |

Abbreviations: NIHSS, National Institute of Health Stroke Scale; TOAST, Trial of Org 10172 in Acute Stroke Treatment; LAA, large artery atherosclerosis; CE, cardio-embolism; SAO, small artery occlusion; DM, diabetes mellitus; CAD, coronary artery disease; AF, atrial fibrillation; SBP, systolic blood pressure; DBP, diastolic blood pressure; WBC, white blood cell; CRP, C-reactive protein; TBil, Total bilirubin; TC, total cholesterol; TG, total triglyceride; HDL, high density lipoprotein; LDL, low density lipoprotein; apoA, Apolipoprotein A; apoB, Apolipoprotein B; UN, urea nitrogen; UA, uric acid; FBG, fasting blood glucose; GHb, Glycosylated hemoglobin; ALT, Alanine aminotransferase; AST, Aspartate aminotransferase.

p<0.05,*.
