# Supplementary material for: Predictors of early neurological deterioration in patients with acute ischemic stroke
Source: Front Neurol. 2024 Aug 21;15:1433010. doi: 10.3389/fneur.2024.1433010 (PMC11371773; doi:10.3389/fneur.2024.1433010)
Supplement: Supplementary file 3 [file Table_2.DOCX]

Supplementary Table 2 Baseline characteristics of included patients at Fifth Hospital

| Variable | END | Non-END | P |
| --- | --- | --- | --- |
|  | (n=18) | (n=227) |  |
| Age, years | 72.5[64,81] | 67[59,77] | 0.055 |
| Male, n(%) | 11(61.1) | 156(68.7) | 0.505 |
| drinking, n(%) | 2(11.1) | 53(23.3) | 0.231 |
| smoking, n(%) | 7(38.9) | 90(39.6) | 0.949 |
| Baseline NIHSS score | 4.5[2,7] | 4[2,6] | 0.374 |
| TOAST |  |  | 0.103 |
| LAA, n(%) | 10(55.6) | 89(39.2) |  |
| CE, n(%) | 4(22.2) | 23(10.1) |  |
| SAO, n(%) | 3(16.7) | 96(42.3) |  |
| other, n(%) | 1(5.6) | 19(8.4) |  |
| hypertension, n(%) | 17(94.4) | 144(63.4) | 0.008^**^ |
| DM, n(%) | 7(38.9) | 82(36.1) | 0.814 |
| CAD, n(%) | 2(11.1) | 12(5.3) | 0.305 |
| AF, n(%) | 5(27.8) | 31(13.7) | 0.103 |
| anticoagulant, n(%) | 5(27.8) | 20(8.8) | 0.011^*^ |
| SBP, mmHg | 148[140,164] | 140[130,155] | 0.078 |
| DBP, mmHg | 83.5[80,90] | 80[78,90] | 0.137 |
| WBC, 109 | 7.47[6.59,9.47] | 7.02[5.68,8.33] | 0.195 |
| neutrophile, 109 | 5.17[4.26,6.91] | 4.43[3.53,5.52] | 0.146 |
| lymphocyte, 109 | 1.97[1.36,2.23] | 1.74[1.32,2.24] | 0.597 |
| monocyte, 109 | 0.55[0.43,3.7] | 0.64[0.43,5.6] | 0.557 |
| platelet, 109 | 201.5[157,219] | 204[165,241] | 0.597 |
| CRP, mg/L | 2.79[1.64,8.88] | 2[0.63,5.98] | 0.146 |
| TBil, μmol/L | 12.9[10.7,17.1] | 10.7[8.1,15.3] | 0.07 |
| TC, mmol/L | 4.45[3.86,4.88] | 4.16[3.56,4.83] | 0.351 |
| TG, mmol/L | 1.10[0.84,1.89] | 1.32[0.9,1.82] | 0.643 |
| HDL, mmol/L | 1.01[0.94,1.39] | 1[0.85,1.25] | 0.416 |
| LDL, mmol/L | 2.93[2.52,3.44] | 2.72[2.11,3.23] | 0.241 |
| apoA, g/L | 1.27[1.21,1.44] | 1.28[1.13,1.44] | 0.562 |
| apoB, g/L | 0.89[0.69,1] | 0.89[0.74,1.08] | 0.257 |
| UN, μmol/L | 5.52[4.6,7.3] | 5.1[4.2,6.1] | 0.207 |
| UA, μmol/L | 326.3[296,407] | 296[234,363] | 0.132 |
| creatinine, μmol/L | 69[62,77] | 73[61,86] | 0.393 |
| FBG, mmol/L | 5.28[4.92,7.99] | 5.26[4.63,6.82] | 0.518 |
| GHb, % | 5.7[5.4,6.3] | 6[5.5,7.2] | 0.262 |
| ALT, U/L | 14.3[11,17] | 16[12.3,22] | 0.223 |
| AST, U/L | 17.1[14.3,21.4] | 17.9[14.8,22.3] | 0.645 |

Abbreviations: NIHSS, National Institute of Health Stroke Scale; TOAST, Trial of Org 10172 in Acute Stroke Treatment; LAA, large artery atherosclerosis; CE, cardio-embolism; SAO, small artery occlusion; DM, diabetes mellitus; CAD, coronary artery disease; AF, atrial fibrillation; SBP, systolic blood pressure; DBP, diastolic blood pressure; WBC, white blood cell; CRP, C-reactive protein; TBil, Total bilirubin; TC, total cholesterol; TG, total triglyceride; HDL, high density lipoprotein; LDL, low density lipoprotein; apoA, Apolipoprotein A; apoB, Apolipoprotein B; UN, urea nitrogen; UA, uric acid; FBG, fasting blood glucose; GHb, Glycosylated hemoglobin; ALT, Alanine aminotransferase; AST, Aspartate aminotransferase.

p<0.05,*; p<0.01,**.
