# Supplementary material for: Predictors of early neurological deterioration in patients with acute ischemic stroke
Source: Front Neurol. 2024 Aug 21;15:1433010. doi: 10.3389/fneur.2024.1433010 (PMC11371773; doi:10.3389/fneur.2024.1433010)
Supplement: Supplementary file 4 [file Table_3.DOCX]

Supplementary Table3 Baseline characteristics of the external validation cohorts

| Variable | Shidong Hospital cohort | Fifth Hospital cohort |
| --- | --- | --- |
|  | (n=154) | (n=245) |
| Age,years | 62[53,71] | 68[59,77] |
| Male,n(%) | 113(73.4) | 167(68.2) |
| drinking,n(%) | 65(42.2) | 55(22.4) |
| smoking,n(%) | 69(44.8) | 97(39.6) |
| Baseline NIHSS score | 5[3,10] | 4[2,6] |
| TOAST |  |  |
| LAA,n(%) | 62(40.3) | 99(40.4) |
| CE,n(%) | 32(20.8) | 27(11) |
| SAO,n(%) | 55(35.7) | 99(40.4) |
| other,n(%) | 5(3.2) | 20(8.2) |
| hypertension,n(%) | 66(42.9) | 161(65.7) |
| DM,n(%) | 42(27.3) | 89(36.3) |
| CAD,n(%) | 14(9.1) | 14(5.7) |
| AF,n(%) | 32(20.8) | 36(14.7) |
| Anticoagulant drug,n(%) | 10(6.5) | 25(10.2) |
| SBP,mmHg | 149[134,165] | 140[130,155] |
| DBP,mmHg | 83[76,95] | 80[79,90] |
| WBC,109 | 7.98[6.23,9.46] | 7.04[5.7,8.41] |
| neutrophile,109 | 5.78[4.02,7.43] | 4.48[3.57,5.56] |
| lymphocyte,109 | 1.51[1.14,2.05] | 1.75[1.32,2.24] |
| monocyte,109 | 0.49[0.39,0.6] | 0.64[0.43,5.5] |
| platelet,109 | 189[160,214] | 204[165,241] |
| CRP,mg/L | 1.46[0.71,3.92] | 2[0.64,6] |
| TBil,μmol/L | 15.1[11.7,19] | 10.8[8.2,15.4] |
| TC,mmol/L | 4.92[4.1,5.62] | 4.17[3.57,4.83] |
| TG,mmol/L | 1.23[0.82,1.91] | 1.3[0.89,1.82] |
| HDL,mmol/L | 1.17[0.99,1.32] | 1[0.86,1.25] |
| LDL,mmol/L | 2.79[2.28,3.41] | 2.74[2.12,3.24] |
| apoA,g/L | 1.28[1.13,1.49] | 1.28[1.14,1.44] |
| apoB,g/L | 0.98[0.79,1.14] | 0.89[0.74,1.06] |
| UN,μmol/L | 4.98[4.14,7.35] | 5.1[4.2,6.1] |
| UA,μmol/L | 341[292,402] | 299[242,370] |
| creatinine,μmol/L | 70[61,80] | 73[61,85] |
| FBG,mmol/L | 5.88[5.1,7.1] | 5.26[4.65,6.94] |
| GHb,% | 5.7[5.3,6.4] | 5.9[5.5,7.2] |
| ALT,U/L | 21[16,29] | 15.8[12.3,21.1] |
| AST,U/L | 20[16,25] | 17.9[14.8,22.2] |
| END,n(%) | 16(10.4) | 18(7.3) |

Abbreviations: NIHSS, National Institute of Health Stroke Scale; TOAST, Trial of Org 10172 in Acute Stroke Treatment; LAA, large artery atherosclerosis; CE, cardio-embolism; SAO, small artery occlusion; DM, diabetes mellitus; CAD, coronary artery disease; AF, atrial fibrillation; SBP, systolic blood pressure; DBP, diastolic blood pressure; WBC, white blood cell; CRP, C-reactive protein; TBil, Total bilirubin; TC, total cholesterol; TG, total triglyceride; HDL, high density lipoprotein; LDL, low density lipoprotein; apoA, Apolipoprotein A; apoB, Apolipoprotein B; UN, urea nitrogen; UA, uric acid; FBG, fasting blood glucose; GHb, Glycosylated hemoglobin; ALT, Alanine aminotransferase; AST, Aspartate aminotransferase.

END, early neurological deterioration.
